# The spinal lymphatic system: an emerging pathway bridging fluid homeostasis, immunity, and disease

**DOI:** 10.1038/s41413-026-00508-6

**Published:** 2026-03-05

**Authors:** Yubao Hou, Jianwei Wu, Shuo Yang, Hongwei Wang, Xianghe Wang, Zian Lu, Zhenhao Chen, Jing Feng, Hongli Wang

**Affiliations:** 1 Department of Orthopedics, Huashan Hospital, Fudan University, Shanghai, China; 2 State Key Laboratory of Chemical Biology, Shanghai Institute of Materia Medica, Shanghai, China

**Keywords:** Bone, Neurophysiology

## Abstract

The lymphatic system, traditionally regarded as a unidirectional conduit for interstitial fluid and immune cell transport, has recently been redefined through the discovery of lymphatic networks along the spinal axis. These spinal lymphatic vessels, encompassing the spinal cord, vertebral bones, and intervertebral discs, challenge long-standing anatomical dogmas and introduce new perspectives on the interplay between the central nervous system (CNS) and the vertebral column. This review systematically summarizes the distribution and dual functions of the spinal lymphatic system in regulating cerebrospinal fluid drainage, maintaining tissue homeostasis, and mediating immune responses. Furthermore, we highlight emerging evidence linking spinal lymphatic dysfunction to spinal pathologies, neurological disorders, and vertebral degeneration. Based on these findings, we propose that the spinal lymphatic system constitutes a previously underappreciated pathway integrating spinal cord and vertebral physiology, with potential implications for both disease progression and therapeutic intervention. While research on the cranial lymphatic system has rapidly advanced, the spinal lymphatic system remains comparatively underexplored. We hope this review will catalyze further investigation into spinal lymphatic biology and inform the development of novel therapeutic strategies targeting spinal and neurological diseases.

## Introduction

The lymphatic vessels (LVs), first noted by Hippocrates in the fourth century BC, form a network distributed throughout most organs. Over the past four centuries, progressive discoveries have expanded our understanding of their anatomy and function.^1^ Particularly during the past century, advances in electron microscopy, live imaging, and isolated lymphatic techniques have provided profound insights into LV structure and dynamics.^2–4^

Lymphatic endothelial cells (LECs) constitute the lining of LVs and characteristically express canonical markers including *Prox1*, podoplanin (PDPN), LYVE-1, and VEGFR-3.^5^ According to the organization of LEC junctions, smooth muscle coverage, and valve presence, LVs are categorized into initial, pre-collecting, and collecting vessels,^6–8^ each exhibiting distinctive LEC morphology and marker expression.^5,7,9–12^ Capillary LECs in peripheral LVs display unique oak leaf-like or lobate-shaped morphologies,^13^ while collecting vessel LECs exhibit elongated shapes. Modern understanding revises the traditional view of purely button- and zipper-like junctions between LECs. Instead, curvilinear and double-contoured junctional patterns also predominate, maintained by dynamic cytoskeletal regulation that balances barrier integrity with permeability.^6,13^

Traditionally, LVs are recognized for their roles in transporting interstitial fluid, immune cells, and lipids, thus participating in immune surveillance and tumor metastasis.^14^ The lymphatic vascular system absorbs interstitial fluid via initial LVs and directs it through pre-collectors and collecting vessels to lymph nodes (LNs).^5,14^ Increasingly, studies highlight organ-specific heterogeneity in LVs and their contributions to diverse physiological and pathological processes,^5,15^ including obesity,^16^ cardiovascular disease,^17^ and glaucoma.^18^

This conventional understanding has been reshaped by the discovery of LVs in the central nervous system (CNS), an area once considered alymphatic.^19–21^ The glymphatic system, facilitating cerebrospinal (CSF) and interstitial fluid exchange through perivascular pathways, has been well characterized in the brain parenchyma.^19,22^ Moreover, systemic hyperosmolality has been shown to enhance drug delivery via periarterial CSF influx into the spinal cord, suggesting an active, glymphatic-like clearance mechanism within the spinal axis.^23–25^ The identification of cranial meningeal lymphatic vessels (mLVs) has redefined CNS immunity and fluid regulation.^20,21^ While cranial mLVs have been widely studied in health and disease,^26,27^ the spinal meningeal lymphatic vessels (smLVs) remain comparatively understudied.^22^

Recent findings reveal that lymphatic networks also extend through the spinal cord, vertebral column, and surrounding paravertebral tissues, contributing to fluid drainage and immune regulation.^28,29^ Our recent work identified LVs in healthy intervertebral discs (IVDs), providing novel insight into the pathogenesis of IVD degeneration.^30^ Furthermore, evidence of LVs in bone suggests their participation in bone metabolism and remodeling.^28,31,32^

Collectively, these discoveries overturn the long-held assumption that perispinal tissues lack lymphatic vasculature. Instead, they reveal an extensive and functionally integrated lymphatic system linking the spinal cord, vertebrae, and IVDs. This overlooked network may play crucial roles in maintaining spinal homeostasis and mediating crosstalk among spinal components. The present review summarizes the distribution and functions of LVs within the spinal region and discusses their involvement in disease progression, highlighting potential therapeutic opportunities for spinal cord injury (SCI), vertebral degeneration, and inflammatory disorders.

## Spinal meningeal LVs (smLVs)

### Development and distribution

Comprehensive characterization of smLVs relies on the identification of reliable markers and refined methodological approaches. Similar to cranial mLVs, smLVs express canonical lymphatic markers such as Prox1, PDPN, and LYVE-1^20,21^ providing a consistent molecular basis for their detection and analysis. Using these markers, multiple experimental strategies have been developed to investigate smLV architecture and function at genetic, protein, and imaging levels. For example, transgenic reporter mice expressing GFP under the Prox1 promoter enable precise visualization of lymphatic distribution,^33^ while Cre-loxP-based genetic manipulations permit temporal and cell type-specific modulation of LEC activity.^28,34^ Immunolabeling targeting proteins such as VEGFR-3, coupled with ligand or inhibitor-based approaches, has further expanded the capacity to probe smLV signaling and remodeling.^35^ In addition, tracer-based assays and advanced in vivo imaging modalities have been employed to map smLV drainage routes and dynamic behavior.^35,36^ Continued progress in molecular and imaging technologies will undoubtedly enhance accessibility to spinal lymphatic research, paving the way toward a more comprehensive understanding of smLV physiology and its relevance to spinal and neurological disorders.

With these advancements, mLVs are becoming increasingly well characterized.^33^ Studies have revealed that similar to peripheral lymphangiogenesis, mLVs also undergo sprout extension and fusion of cell clusters, with their development and maintenance dependent on VEGF-C-VEGFR3 signaling.^33^ Visualizing lymphatic development in mice has demonstrated that peripheral LVs begin to develop between embryonic day 9.5 and embryonic day 10^37^. Intracranial mLVs initiate their development postnatally, significantly later than peripheral LVs.^38^ Observable smLVs emerge from postnatal day 4 and connect with intracranial mLVs by postnatal day 8.^33^ Nevertheless, research on the development of smLVs is limited, and the origin of meningeal LECs still remains poorly understood.^37^

**Fig. 1:**
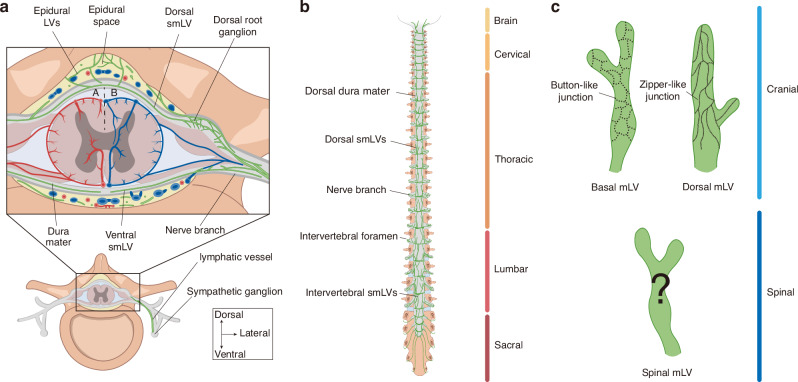
Overview of lymphatic vessels in spinal dura mater and paravertebral space. **a** Cross-sectional view of spinal meningeal lymphatic vessels (smLVs) in the spinal dura mater and corresponding epidural spaces, shown with arterial (A) and venous (B) distributions. Dorsal LVs exhibit greater density and crosslinking compared to ventral vessels. Ventral and dorsal smLVs and epidural LVs converge near the dorsal root ganglion (DRG), forming an encircling network. This network exits along the nerve branch and shows a connection with the sympathetic ganglion. **b** SmLV within the dorsal spinal dura mater. SmLVs are segmentally distributed along the dorsal dura mater and exit the spinal canal laterally together with nerve roots. They are more abundant in the thoracic and lumbar regions, and caudal smLVs appear larger and more rounded. **c** Comparison of cranial and spinal mLV junction patterns. While lymphatic endothelial cells (LECs) in cranial mLVs exhibit distinct button- and zipper-like junctional patterns, the junctional morphology of smLVs remains unclear. Color coding: Brown, vertebra; Dark brown, nerve; Gray, dura mater; Green, lymphatic vessel; Red, artery; Blue, vein. LV lymphatic vessel, smLV spinal meningeal lymphatic vessel, DRG dorsal root ganglion, LECs lymphatic endothelial cells

To investigate the distribution of smLVs, research utilizes immunostaining to label lymphatic markers such as CD31, LYVE-1, and Prox1. The meninges covering the vertebrae and IVDs in mice with removed spinal cords have been examined. Generally, smLVs show clustered and segmentally high distributed along dura mater.^35,39^ Lower smLV density has been identified in the cervical and sacral regions, with higher expression in the thoracic and lumbar regions.^39^ Research has observed that the smLVs on the ventral side are not connected at the midline.^33^ And on dorsal side, they exhibit more densities, crosslinks, and connections to cerebral cisterns^33,35^(Fig. 1a, b). During development, the dorsal caudal lymphatic distribution area became larger and more rounded to accommodate spinal elongation^33^ (Fig. 1b). Additionally, dorsal dural LVs are observed to localize around the dorsal root ganglia (DRG) and spinal nerve branches, exiting the spinal canal laterally alongside nerve roots^35^ (Fig. 1a, b). These segmental vessels are connected by intervertebral smLVs and show connection to the epidural conduits, suggesting potential convergence points for peripheral lymph and CSF drainage.^35^ Besides, anatomical investigations have identified smLVs extending from DRG to sympathetic ganglia, indicating a potential anatomical link between the autonomic nervous system and spinal lymphatic network^35^ (Fig. 1a). Furthermore, Prox1 and LYVE-1 staining revealed that these vessels in the thoracic segment exhibit a staining pattern of short, discontinuous lines attached to vascular vessels.^39^ This finding suggests a distributional and functional linkage with the vascular system, which needs further investigation.

Collectively, these studies demonstrate the existence of LVs in the spinal dura mater. SmLVs envelop the DRG and exit the spinal canal alongside nerve roots and blood vessels. Further research is required to determine the relationships between anatomical distribution, LV density, and drainage function.

### Drainage function of smLVs across spinal segments

For nearly a century, CSF outflow pathways within the CNS have been extensively investigated.^40–42^ MLVs are now recognized as important conduits that facilitate waste clearance not only from the dura and subdural spaces^43–45^ but also from the CNS to peripheral lymphatic systems, working in concert with the glymphatic system.^20,21^ While intracranial CSF drainage routes, particularly via the cribriform plate and along olfactory nerves to the deep cervical lymph nodes (CLNs), are well established,^6,40,46,47^ the existence and mechanisms of spinal CSF outflow remain less clearly defined and somewhat controversial.^42^

Early investigations estimated that spinal CSF outflow contributes approximately 16%–25% of total CSF drainage.^36,48,49^ However, the specific pathways and relative importance of this drainage remain subjects of debate. Some studies argued that spinal CSF drains predominantly through venous pathways.^50^ Specifically, in studies on human cadavers, Kido et al. identified spinal arachnoid villi and found that perfused dye could enter the lumen of venous sinuses,^50^ indicating the venous drainage. Meanwhile, others have proposed that lymphatic drainage becomes significant only under pathological conditions.^42,51,52^ In supporting this, Galkin et al. surgically separated the cribriform plate in dogs and observed that India ink tracers drained through the lumbosacral region. This finding suggests that such drainage becomes prominent only when intracranial outflow pathways are obstructed or when tracers are delivered under high-pressure injection conditions.^51^

Nevertheless, a major limitation is that the connection between CSF and venous sinuses has been demonstrated only in cadavers, without in vivo confirmation.^50^ Moreover, the tracers used in earlier studies may not accurately reflect physiological CSF dynamics.^51^ Recent studies have overcome these challenges by leveraging the advent of modern technologies, such as light-sheet fluorescence microscopy (LSFM), confocal imaging, and whole-tissue immunolabeling. These methods offer markedly improved detection sensitivity.^35,36,53–55^ Using these new tools, researchers injected novel tracers (e.g., OVA-A555 and LYVE-1 antibodies) into the subarachnoid space in mice and successfully tracked the draining pathway, identifying definitive CSF-draining pathways through smLVs in healthy mice.^35,36,53–55^ These studies have established smLV-mediated CSF drainage as a physiological process rather than a pathological artifact. And this drainage mechanism is proposed to be particularly significant in upright species, including humans.^36^ Importantly, the drainage capacity of smLVs varies significantly across spinal segments,^35,36,56^ likely reflecting adaptations to local anatomical and physiological demands.

In the cervicothoracic region, injection of ultrafine carbon particles into the subarachnoid space results in broad labeling of LVs and LNs within the cervical and thoracic epidural tissues surrounding nerve roots.^48^ Following intracerebroventricular injection of quantum dots, fluorescent signals rapidly appear in nasal-associated lymphoid tissue, CLNs, and thoracic LNs.^56^ Over time, signals extend along DRG and thoracolumbar nerves, accumulating in lumbar and sacral LNs. Complementary experiments using OVA-A555 injection into thoracolumbar parenchyma, combined with iDISCO+/LSFM and confocal microscopy, revealed tracer deposition in thoracic and lumbar epidural spaces and smLVs, with subsequent detection in paravertebral lymph vessels and mediastinal LNs within 15 min.^35,55^ In parallel, other studies have demonstrated that under physiological conditions, CSF flows through the subarachnoid space and central canal toward the sacrococcygeal region.^36^ After intracisternal magna injection of OVA-A555, strong tracer signals were detected in the sacrococcygeal epidural space and along the vertebral canal, sciatic nerve, lumbar, and renal LNs.^36,55^ Together, these findings indicate that tracers injected into the subarachnoid space distribute broadly along the spinal axis following CSF flow,^36,56^ whereas tracers delivered into the spinal cord parenchyma are preferentially drained through LVs at the corresponding spinal segment, particularly evident in thoracolumbar regions.^35^ Further experiments are needed to clarify how the injection site and segmental anatomy determine lymphatic drainage routes.

Furthermore, among spinal levels, sacrococcygeal smLVs exhibit the highest drainage efficiency, although their absolute outflow volume remains lower than that of cranial mLVs.^36,55^ The relatively limited drainage capacity of other spinal segments may underlie the difficulty in measuring spinal CSF egress. Collectively, these findings establish smLVs as an essential clearance route under both physiological and disease conditions.

Despite this progress, the precise mechanisms governing CSF transport from the subarachnoid space to smLVs remain unresolved.^42^ Traditionally, in cranial mLVs, button-like endothelial junctions facilitate CSF uptake, whereas zipper-like junctions mediate collective drainage.^6,57^ Although cranial mLVs are mostly confined to the dura mater, evidence suggests potential connections with CSF spaces; however, the extent of their penetration through the arachnoid remains uncertain.^44,45^ Likewise, intracranial arachnoid granulations act as specialized lymphatic structures,^57,58^ yet corresponding spinal counterparts are poorly characterized. Microstructural studies delineating the junctional architecture of smLVs in distinct segments are lacking (Fig. 1c), limiting our ability to distinguish capillary from collecting lymphatic domains. Although postmortem observations have identified arachnoid granulations projecting toward the spinal dura, particularly near nerve roots,^50,59^ spinal arachnoid villi remain incompletely studied.^50,60^ An additional hypothesis proposes that a modified region at the subarachnoid angle, where the arachnoid membrane thins and tight junctions are sparse, may act as an entry site for CSF into smLVs.^61^

Overall, the incomplete understanding of smLV microanatomy continues to hinder functional interpretation. The technical difficulty of preserving intact spinal meninges and precisely quantifying minute CSF fluxes further complicates analysis.^22^ Future studies should aim to define the microstructural organization and dynamic properties of the subarachnoid angle, potentially a key interface for smLV-mediated CSF exchange. Integration of advanced imaging techniques with molecular and single-cell analyses is expected to provide critical insights into spinal CSF circulation and its regulation of CNS homeostasis.

### Modulation of smLVs drainage efficiency

**Fig. 2:**
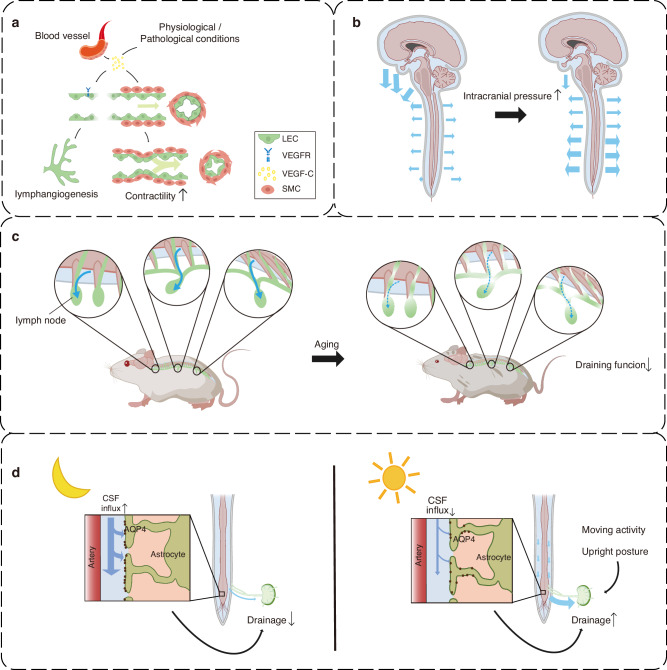
Regulation of smLV drainage efficiency. **a** VEGF-C secreted by smooth muscle cells (SMCs) surrounding blood vessels and other local sources enhances drainage efficiency by promoting lymphangiogenesis and increasing lymphatic endothelial cell (LEC) contractility. **b** Elevated intracranial pressure reduces cervical lymphatic drainage but augments drainage from lower spinal meningeal lymphatic vessels (smLVs), reflecting a compensatory redistribution of CSF outflow. **c** Aging markedly decreases drainage efficiency along the spinal cord, accompanied by diminished lymphatic density and functional decline. **d** Circadian rhythm critically modulates smLV drainage, with efficiency peaking during the active (daytime) phase and declining at night. These fluctuations are influenced by glymphatic activity, body posture, and locomotor patterns. smLV spinal meningeal lymphatic vessel, SMC smooth muscle cell

The drainage efficiency of smLVs varies considerably under physiological and pathological conditions. Among the regulatory pathways, VEGF-C/VEGFR-3 signaling shows the strongest association with enhanced lymphatic drainage. This pathway promotes lymphangiogenesis and stimulates the contractile activity of smooth muscle cells (SMCs) surrounding collecting vessels, thereby accelerating lymph transport.^33,62^ Notably, SMCs surrounding blood vessels act as a major paracrine source of VEGF-C, sustaining LV growth and maintenance^33^ (Fig. 2a). Intracranial pressure is another key determinant of smLV drainage. Elevated intracranial pressure reduces cervical lymphatic drainage but simultaneously enhances spinal meningeal lymphatic flow^52,63^ (Fig. 2b). When cranial outflow routes are obstructed, smLVs exhibit compensatory upregulation, suggesting a dynamic adaptive mechanism.^63^

Aging profoundly impairs meningeal lymphatic function. Studies show that aged mice display markedly reduced tracer clearance through jugular and mandibular LNs,^53^ accompanied by a progressive decline in thoracic and lumbar clearance rates^64^ (Fig. 2c). Anatomical evidence also suggests potential interactions between smLVs and sympathetic ganglia. Although autonomic dysfunction is known to compromise peripheral lymphatic activity,^65^ whether sympathetic signaling modulates smLV contractility or drainage remains unexplored.

Circadian rhythms tightly influence CNS fluid homeostasis and lymphatic function, with implications for neurological and cardiovascular disorders.^66,67^ smLV drainage exhibits circadian oscillations that inversely correlate with glymphatic influx dynamics.^66,68^ In rodents, glymphatic activity peaks during the resting (light) phase and diminishes during wakefulness, paralleling suppression of periarterial influx.^66,68,69^ Conversely, smLV-mediated CSF outflow increases during wakefulness, showing enhanced tracer spread throughout the spinal cord. These opposing cycles suggest a coordinated alternation between glymphatic inflow and lymphatic outflow.^66,68–71^ Mechanistically, AQP4 redistribution, astrocytic morphology, vascular pulsation, and respiratory-driven CSF oscillations may contribute to these temporal variations.^66,68,69,71,72^ Locomotor activity and posture also modulate spinal lymphatic flow, with upright positions enhancing outflow in the lumbosacral region through gravitational and anatomical adaptations.^56^ Experimental interventions that entrain circadian glymphatic-lymphatic coupling, such as melatonin supplementation or blue light therapy to reinforce sleep-wake alignment, are under evaluation for promoting brain waste clearance,^73^ though their effects on smLV drainage remain to be determined. Further research should also clarify how physical activity and posture optimize smLV flow across circadian phases (Fig. 2d).

In summary, the spinal meningeal lymphatic system demonstrates remarkable functional plasticity and compensatory capacity in response to fluctuations in CNS fluid dynamics. This adaptability may provide neuroprotective benefits in disorders characterized by impaired CSF clearance. Elucidating the molecular and physiological determinants of smLV modulation will be critical for identifying therapeutic strategies to restore or enhance spinal lymphatic drainage.

### Immunological functions and modulations of smLVs

Historically, the CNS has been viewed as an immune‑privileged organ, largely isolated from peripheral surveillance due to the blood-brain barrier.^26^ With the discovery of smLVs, this perspective has shifted. The spinal cord is now regarded as an “immunologically quiescent” region, equipped with resident immune cells that can mount adaptive responses when necessary.^74,75^ Immune cells have been identified within smLVs, implicating these vessels as conduits between CNS and peripheral immunity.^21^ The dura mater, in particular, serves as a dynamic interface where smLVs coordinate communication between the CNS and peripheral immune systems.^76,77^

Under steady‑state conditions, the dura mater hosts macrophages, dendritic cells, innate lymphoid cells, mast cells, neutrophils, B cells, and T cells.^76^ Among them, macrophages dominate numerically and functionally, contributing to tissue remodeling, angiogenesis, and repair.^22^ They intimately associate with smLVs,^55,78^ and tracer studies demonstrate that intrathecal injections at the thoracolumbar level yield macrophage‑associated signal in meninges and lumbar LNs.^55^ These findings suggest that smLV‑mediated drainage establishes bidirectional immune communication between spinal and peripheral compartments.^55^ Moreover, macrophages produce lymphangiogenic factors essential for smLV growth; their depletion significantly suppresses meningeal lymphangiogenesis, particularly during inflammation, SCI, or aging.^78^

Beyond macrophages, adaptive immune cells are integral to spinal meningeal immunity. B cells constitute roughly 25%–30% of immune cells in the dura, while CD4⁺ T cells account for approximately 5%.^79^ Dura‑associated lymphoid tissue (DALT), functionally analogous to mucosa‑associated lymphoid tissue, contains proliferating B cells, T follicular helper cells, and plasma cells localized around dural sinuses.^80^ These structures mediate humoral immune responses to both peripheral and CSF antigens and can generate germinal centers independent of systemic immunity.^80^ DALT may also serve as a precursor to tertiary lymphoid structures (TLS), which emerge during chronic inflammation and have been detected in multiple sclerosis (MS), SCI, and degenerative spine disease.^81–83^ TLS can exert dual effects, providing local immune regulation or amplifying pathological inflammation as shown in experimental autoimmune encephalomyelitis (EAE).^81^ Most meningeal CD4⁺ T cells derive from circulation (~20%), whereas antigen‑presenting macrophages and B cells likely originate from adjacent cranial and vertebral bone marrow.^82,84^ The contribution of smLVs to DALT or TLS formation remains poorly defined.

In the cranium, mLVs facilitate antigen and immune cell drainage to deep CLNs, activating and maturing T and B cells and promoting antibody production.^45,85,86^ This process depends on the CCR7-CCL21 chemokine axis,^22,45^ and is further shaped by MHC‑II‑bound self‑peptides along the CNS-lymphatic continuum.^87^ Dysfunctional mLVs lead to lymphatic regression and exaggerated meningeal immune activation,^76,88^ underscoring the delicate balance between waste clearance and immune stimulation. By analogy, smLVs are hypothesized to serve parallel roles in the spinal compartment,^33^ draining antigens and immune mediators from the spinal microenvironment to regional nodes such as the lumbar and iliac LNs.^36,52^ However, the molecular pathways governing smLV‑mediated immune activity and their contributions to spinal immune homeostasis and pathology remain undefined.

Resident spinal glia also participates in immune-lymphatic crosstalk. Microglia secrete VEGF‑C, which binds VEGFR‑3 to promote lymphangiogenesis while concurrently inhibiting autophagy, thereby sustaining the M1 phenotype.^89^ This autocrine VEGF‑C loop reinforces microglial activation yet simultaneously enhances lymphatic clearance of inflammatory mediators, mitigating secondary injury and facilitating neurological recovery.^78,89^ Whether oligodendrocyte-lymphatic interactions observed in cranial meninges extend to smLVs remains to be determined.^88^

Collectively, smLVs constitute an immunological bridge linking the spinal cord to peripheral lymphatic networks. Although current understanding lags behind that of cranial mLVs, elucidating the mechanisms by which smLVs regulate immune surveillance, antigen transport, and local inflammation may reveal novel therapeutic strategies for spinal cord and neuroinflammatory diseases.

### SmLVs in disease pathophysiology and therapeutic potential

The contribution of smLVs to disease pathophysiology is multifaceted and context-dependent, reflecting their dual roles in CSF clearance and immune regulation. These two functions frequently intersect during disease progression.^35,45,90^ In spinal and neurological disorders, alterations in lymphatic drainage capacity, endothelial proliferation, and vessel remodeling collectively influence local inflammation, edema, and tissue repair.^78,89,91^ Increasing evidence suggests that impaired lymphatic function exacerbates neuroinflammation and accelerates disease progression.^92–94^ Conversely, excessive or aberrant lymphangiogenesis may facilitate maladaptive immune activation or fibrosis, underscoring the need for tightly regulated lymphatic homeostasis.

#### Multiple sclerosis

MS is a chronic autoimmune demyelinating disorder of the CNS characterized by inflammatory destruction of myelin and axons in both the brain and spinal cord.^95^ Its pathological hallmark is the perivenular inflammatory lesion enriched with CD8⁺ T cells, leading to oligodendrocyte injury, demyelination, and progressive neurodegeneration.^96^ The generation of B cell-derived anti-myelin antibodies, which is facilitated by the formation of TLS, also plays a significant role in the pathological process.^81,95^

##### Spinal stenosis and impaired lymphatic dynamics in the pathogenesis of MS

**Fig. 3:**
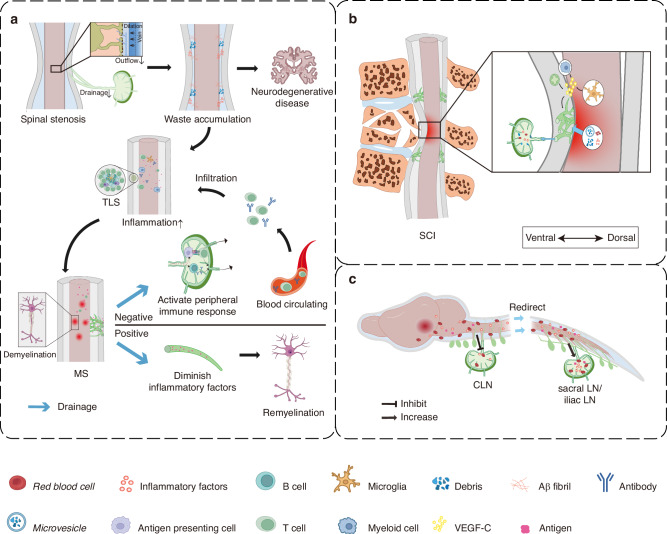
Spinal meningeal lymphatic vessels (smLVs) in disease pathophysiology. **a** Spinal stenosis may interfere with the coordinated function of the glymphatic system and spinal meningeal lymphatic vessels (smLVs), leading to impaired central nervous system (CNS) waste clearance. The clearance deficit is hypothesized to foster a pro-inflammatory microenvironment, thereby increasing susceptibility to neurodegenerative and demyelinating disorders, such as Multiple Sclerosis (MS). MS frequently features tertiary lymphoid structures (TLS) within the meninges. Enhanced smLV drainage in MS may both exacerbate autoimmune responses and aid in inflammatory resolution, reflecting a context-dependent dual role. **b** Spinal cord injury (SCI) induces pronounced lymphangiogenesis in the meninges, facilitating immune factor drainage and mainly contributing to improved recovery outcomes. **c** In cranial or upper spinal cord diseases, such as stroke and tumor, smLVs help remove erythrocytes and other detrimental substances from the cerebrospinal fluid (CSF) more efficiently. While the observation of suppressed activity in cervical lymph nodes (CLNs), contrasted with the comparatively unaffected state of lower smLVs, suggests a potential redirection of waste drainage more towards the caudal regional LNs, such as sacral and iliac LNs. smLV spinal meningeal lymphatic vessel, MS multiple sclerosis, SCI spinal cord injury, LN lymph node, CLN cervical lymph node, CSF cerebrospinal fluid

Spinal stenosis, defined by the narrowing of the spinal canal, is increasingly recognized as a condition that may influence not only neural and vascular integrity but also the spinal lymphatic network.^97,98^ Clinical imaging studies demonstrate that MS lesions frequently colocalize with regions of moderate to severe spinal stenosis,^91^ suggesting a mechanistic association. Stenosis decreases venous and spinal cord compliance, inducing venous dilation and venous reflux, which are hemodynamic changes also observed during MS progression.^97,98^ These vascular alterations likely impair glymphatic flow due to shared anatomical pathways.^97,98^ Supporting this concept, MRI and near‑infrared imaging reveal that CSF movement within the spinal subarachnoid space and its influx into the parenchyma are reduced in early MS.^99,100^ This impaired CSF flow precedes immune infiltration and myelin damage.^100^ Because cortical glymphatic dysfunction has been linked to reduced meningeal lymphatic drainage,^101^ and given the proximity of spinal lymphatics to regions of stenosis,^35,102^ it is plausible that structural compression or venous congestion in stenosis disrupts smLV‑mediated drainage. Such disruption may diminish CSF outflow, exacerbate waste accumulation, and accelerate cortical demyelination, potentially acting as an upstream etiological factor in MS^36,93,103^ (Fig. 3a).

Although altered CSF and glymphatic flow have been consistently documented in MS,^97–100^ direct experimental evidence linking spinal stenosis-induced smLV dysfunction to MS pathogenesis remains limited. We propose that venous dilation, impaired CSF dynamics, and anatomic deformation may collectively drive smLV dysfunction, amplifying inflammatory demyelination. Future investigations employing in vivo imaging, tracer assays, and genetic or surgical modulation of smLVs are needed to test whether compromised spinal lymphatic drainage causally contributes to neuroinflammatory progression in MS.

##### The controversial roles of smLVs throughout MS progression

The overall impact of smLVs in MS remains contentious, with evidence supporting both pathogenic and protective effects. On one hand, several studies suggest that smLVs promote autoimmune activation by draining CNS‑derived antigens to peripheral LNs,^81^ thereby facilitating antigen presentation and T‑cell priming.^81^ In EAE, a widely used MS model, smLVs are associated with accelerated disease progression.^45,46^ Their immune‑surveillance function may amplify inflammation and demyelination by enhancing antigen drainage.^45,46,104^ Supporting this, resection of lumbar LNs that drain the spinal cord alleviates EAE pathology and reduces T‑cell proliferation, highlighting a stronger neuroinflammatory load in spinal versus cervical lymphatic circuits.^104^ Similarly, lysophosphatidylcholine (LPC)‑induced focal demyelination triggers VEGF‑C‑dependent lymphangiogenesis around vertebral smLVs, increasing immune infiltration via VEGFR‑3 signaling.^35^ Inhibition of VEGFR‑3 with MAZ51 diminishes lymphatic expansion, demyelination, and CD4⁺ T‑cell accumulation,^35,46^ while photodynamic ablation of meningeal lymphatics delays EAE onset.^45^ Together, these findings argue for a pro‑inflammatory role of smLVs during active disease.

Conversely, other reports challenge this view, indicating that smLVs may play neutral or even beneficial roles in MS.^34,88,105^ Across multiple EAE models, including active, adoptive transfer, and relapsing-remitting forms, smLV ablation or VEGFR‑3/VEGF‑C blockade did not exacerbate disease severity or alter pathogenic T‑cell trafficking.^34,45,105^ Morphological and transcriptomic analyses also show no major lymphatic remodeling during EAE,^34,45,105^ and inflammation within the spinal dura is considerably milder than in the leptomeninges.^34,105^ Moreover, studies in cranial mLVs demonstrate that lymphatic impairment hinders remyelination and worsens recovery after demyelinating injury.^88^ Ablation of mLVs during the repair phase reduces mature oligodendrocyte survival and delays myelin regeneration, suggesting that intact lymphatic function may facilitate resolution^88^ (Fig. 3a). Clinically, reduced VEGF‑C concentrations in CSF of MS patients, particularly after relapses, further imply that lymphatic insufficiency may correlate with disease activity.^88^(Table 1).

These apparently conflicting findings likely arise from methodological and model‑specific differences. Photodynamic ablation using Visudyne may cause non‑specific immune suppression, confounding interpretation.^34^ The VEGFR‑3 inhibitor MAZ51 exhibits off‑target inhibition of VEGFR‑2-mediated angiogenesis,^93^ while incomplete smLV ablation (e.g., LN excision only) or compensatory drainage from adjacent spinal regions may obscure effects.^33,36^ Additionally, smLV function may differ across MS stages, exerting pro‑inflammatory effects during acute phases but aiding debris clearance and repair during remission.^95^ Therefore, precise spatiotemporal mapping of smLV activity in well‑controlled models is crucial.

Future research should aim to dissect the stage‑dependent and regional functions of smLVs using advanced imaging, conditional knockout strategies, and longitudinal EAE paradigms. Establishing standardized approaches to manipulate smLVs will help resolve current discrepancies and clarify whether targeting spinal lymphatic dynamics can offer therapeutic benefit in MS and related demyelinating disorders.

**Table 1:** Summary of studies investigating the roles of cranial and spinal mLVs in demyelination models

Ref	Model	Key intervention	Location	Key outcome measures	Main conclusion	Proposed role in MS
**Region involved: cranial (without verifying in the spinal cord)**						
Louveau et al.^45^	EAE mice^a^	Visudyne-photodynamic lymphatic ablation	Cranial dura	EAE clinical score, T-cell infiltration	Ablation of meningeal lymphatics delayed disease onset and reduced severity	Aggressive
Hsu et al.^46^	EAE mice^a^	MAZ51 lymphatic ablation, KikGR photoconversion, Evans Blue	Cribriform plate	MRI signals, EAE clinical scores	Neuroinflammation promotes lymphangiogenesis, which enhances CNS antigen drainage and T-cell priming	Aggressive
das Neves et al.^88^	Cuprizone-induced demyelination in mice	VEGF-C/D trap, genetic lymphatic ablation	Cranial dura	Oligodendrocyte and MBP levels, immune profiling	Impairment of meningeal lymphatic function worsens demyelination and reduces oligodendrocyte survival.	Protective
**Region involved: containing the spinal cord**						
van Zwam et al.^104^	EAE^a^ mice	Surgical removal of lymph nodes	Spinal cord	EAE clinical score	Removal of lumbar lymph nodes reduced relapse severity	Aggressive
Jacob et al.^35^	LPC-induced demyelination mice	LPC injection, immunofluorescent	Thoracolumbar spinal cord	Immune cell infiltration	Lymphangiogenesis and immune cell infiltration increase in demyelination	Aggressive
Fournier et al.^99^	PLP-induced EAE mice	Tracer (indocyanine green/Dotarem) injection	Spinal cord	MRI signal	CSF circulation reduces in the spinal cord parenchyma, which may impair waste clearance in the spinal cord	Protective^b^
Merlini et al.^105^	EAE mice and rat^a^	VEGF-C/D trap; AAV-mediated VEGF-C expression	Cranial and spinal dura	Immune cell infiltration, antigen presentation	Inflammation within the spinal dura mater is less severe than in the leptomeninges and spinal parenchyma	None
Li et al.^34^	EAE mice^a^	Pharmacological, genetic lymphatic ablation;	Cranial and spinal dura	Immune cell infiltration, EAE clinical scores	Blocking meningeal lymphatic function had no significant effect on neuroinflammation or clinical severity of EAE	None
Xin et al.^100^	EAE mice^a^	Fluorescent tracer (P40D800) injection	Cribriform plate; spinal cord	Fluorescent intensity	Although CSF circulation reduces in the spinal cord, no significant change in the outflow of CSF to the lymph nodes	None^b^

*MS* multiple sclerosis, *mLV* meningeal lymphatic vessel, *EAE* experimental autoimmune encephalomyelitis, *MBP* myelin basic protein, *LPC* lysophosphatidylcholine, *CSF* Cerebrospinal fluid, *MOG* myelin oligodendrocyte glycoprotein, *CFA* complete Freund's adjuvant, *PTX* pertussis toxin

^a^EAE mice are usually induced by MOG_35−55_, CFA, and PTX

^b^Proposed by us with no direct evidence in text

#### Dementia

Neurodegenerative disorders, particularly Alzheimer’s disease and Parkinson’s disease, have been intensively investigated in relation to cranial mLVs.^26,103,106^ Accumulating evidence suggests that dysfunction of the glymphatic and meningeal lymphatic systems impairs the clearance of neurotoxic metabolites and contributes to cognitive decline.^102^ Cranial mLVs, working synergistically with the glymphatic network, play a pivotal role in removing metabolic waste, including β‑amyloid, tau, and inflammatory mediators, from the CNS.^26,106^ Defective mLV‑mediated drainage accelerates waste accumulation, neuroinflammation, and progressive neurodegeneration.^106^

Although the role of smLVs in dementia remains largely unexplored, their anatomical and functional parallels with cranial mLVs suggest that they may similarly influence disease progression. SmLVs drain interstitial fluid and solutes along the spinal axis,^35,36^ and disruptions in these pathways may compromise overall CSF circulation and metabolic clearance. Lumbar spinal stenosis, a condition that constricts both vascular and lymphatic outflow, has been identified as an independent risk factor for dementia.^102,107^ This pathology likely disturbs not only neural conduction but also vascular and lymphatic homeostasis, echoing the mechanistic links observed between MS and spinal stenosis.^102^ Such impairment may reduce waste clearance through spinal glymphatic and lymphatic routes, promoting accumulation of Aβ and other neurotoxic molecules and thereby contributing to cognitive deterioration^102^ (Fig. 3a).

Future studies should determine whether common spinal pathologies, such as stenosis or IVD herniation, impede spinal lymphatic drainage and how these alterations affect CNS metabolite clearance. In particular, it will be critical to identify the molecular cargo transported by smLVs and to define how their dysfunction interacts with neurodegenerative cascades. Clarifying these mechanisms may uncover new therapeutic opportunities that target spinal lymphatic circulation to slow or prevent dementia progression.

#### Spinal cord injury

SCI is not a single event but a complex, progressive process involving cascading molecular, cellular, and tissue-level responses.^108^ The primary mechanical insult occurs instantaneously, while the ensuing secondary injury unfolds over minutes to weeks, or even longer, encompassing edema, ischemia, excitotoxicity, inflammation, and glial activation.^109^ Emerging evidence highlights smLVs as critical mediators of post-SCI communication between the CNS and peripheral immune compartments.^26,78^

Following SCI, extracellular vesicles and soluble antigens originating from the injured spinal cord are rapidly drained to paravertebral LNs, demonstrating an active lymphatic connection between the spinal cord and peripheral tissues^110^ (Fig. 3b). In both acute and subacute phases, myeloid cells upregulate lymphangiogenic transcriptional profiles, while microglia secrete elevated levels of VEGF-C, driving robust lymphangiogenesis.^78,89^ These lymphangiogenic signals not only expand the smLV network but also suppress microglial autophagy, sustaining their activated phenotype.^89^ Depletion of macrophages or microglia significantly reduces injury-induced lymphangiogenesis, underscoring their essential role in coordinating LEC proliferation and remodeling.^78^ Correspondingly, smLVs display marked increases in *Vegfc* mRNA expression and proliferative activity at the lesion site, extending beyond their normal perineural distribution.^78,89^ This expansion facilitates fluid clearance and removal of inflammatory mediators, thereby mitigating secondary injury and improving neurological recovery.^78,89^ In support of this, mechanical ligation of smLVs exacerbates edema, cellular degeneration, macrophage infiltration, and disruption of the blood-spinal cord barrier, collectively worsening neurological outcomes.^110^ Similarly, omental transplantation after SCI, which has long been considered beneficial for perfusion, may also owe part of its therapeutic efficacy to the functional contribution of its intact lymphatic network, which alleviates post-traumatic edema.^111,112^

Nevertheless, smLV function after SCI is not uniformly protective. Transcriptomic analyses reveal upregulation of thermosensory and pain-associated genes in LECs,^78^ suggesting that aberrant smLV signaling may contribute to central sensitization and neuropathic pain. Furthermore, smLVs likely participate in the bidirectional immune crosstalk initiated by SCI. On one hand, they transport CNS-derived antigens to draining LNs, facilitating systemic immune activation and leukocyte recruitment into spinal lesions.^76,88,113^ On the other hand, smLVs aid in resolving local inflammation by clearing cytokines, cellular debris, and excess interstitial fluid.^46^ Given that both innate and adaptive immune responses can exert reparative or detrimental effects depending on timing and intensity,^74,109^ the overall impact of smLVs is inherently stage-dependent.

Taken together, smLVs appear to play a dual role in SCI pathology, protective during acute injury through enhanced drainage and resolution of inflammation, yet potentially detrimental during later phases by sustaining immune activation or neuropathic pain. Dissecting the temporal and mechanistic transitions between these states remains a major challenge. Future studies should focus on delineating phase-specific functions of smLVs using longitudinal imaging, genetic tools, and selective interventions to identify therapeutic windows for targeted modulation of spinal lymphatic activity to maximize neurological recovery.

#### Other neurological diseases

##### Stroke

The cranial mLVs exhibit a complex and context-dependent role in stroke progression, balancing beneficial drainage with potentially harmful immune activation. Efficient mLV-mediated clearance is neuroprotective: disruption of these vessels exacerbates injury severity in both transient middle cerebral artery occlusion^114^ and subarachnoid hemorrhage (SAH),^90^ by impairing removal of interstitial solutes and cellular debris. Conversely, excessive immune activation within the CLNs can aggravate secondary inflammation.^113,115,116^ After SAH, LECs within CLNs internalize erythrocytes and promote macrophage-driven inflammatory cascades,^113,115^ ultimately worsening neurological damage. Notably, surgical excision of CLNs, while preserving cranial lymphatics, reduces infarct volume and improves outcomes,^113,115^ highlighting that mLVs may exert divergent effects depending on which component of the lymphatic-immune axis is targeted. These dual roles likely reflect differences in intervention approach and also disease stage. However, during the acute and recovery phases of stroke, the relative contribution of mLVs remains poorly defined. Clarifying how mLVs regulate drainage and immune responses, and determining which of these functions predominates over time, will be essential for therapeutic translation.

Beyond the cranium, mounting evidence suggests that stroke pathology extends into the spinal domain. SAH profoundly impairs cranial lymphatic function but exerts less effect on spinal lymphatic drainage,^117^ drawing attention to the potential compensatory involvement of smLVs. Hemoglobin deposition in sacral LNs following SAH implies an alternative clearance route, albeit less efficient than cervical drainage^117^ (Fig. 3c). Under pathological conditions, this pathway may become activated to facilitate elimination of hemorrhagic cerebrospinal fluid, contributing to post-stroke recovery.^117^ However, direct causal evidence linking smLV dysfunction to worsened stroke outcomes remains lacking. The absence of systematic experimental and clinical studies represents a major knowledge gap. Furthermore, interactions between spinal ischemia or hemorrhage and local smLV function have not been explored. Clarifying whether smLVs participate directly in spinal stroke or serve as secondary compensatory conduits will be crucial for understanding their pathophysiological and therapeutic significance.

##### CNS tumors

In CNS tumors, cranial mLVs play a pivotal role in coordinating anti‑tumor immunity by draining tumor‑associated antigens to deep CLNs, where they promote T‑cell priming and clonal expansion.^85,118^ Disruption or ablation of mLVs compromises antigen clearance, resulting in cerebral edema and impaired anti‑tumor responses.^85,118^ Conversely, enhancing mLV function augments the efficacy of immune checkpoint blockade and radiotherapy, prolongs survival, and induces durable immune memory.^118,119^ Although LVs are traditionally associated with metastasis,^14,26^ current evidence indicates that VEGF‑C does not promote glioma dissemination.^26,85^ In contrast, immune‑enriched meningiomas exhibit elevated lymphatic density and expression of lymphatic markers, suggesting that context‑specific lymphangiogenesis may contribute to tumor progression.^120^ These findings highlight the dual nature of lymphatics in the CNS, facilitating both immune surveillance and, under certain conditions, tumor‑associated remodeling. Future work should clarify whether parenchymal tumor cells access the CSF and delineate their diverse metastatic pathways.^26^

Despite the extensive characterization of cranial mLVs, the role of smLVs in intraspinal tumors remains virtually unexplored.^26,85,121^ In murine glioma models, obstruction of cranial lymphatic outflow reduces total CSF drainage and redirects more tracer flow toward the caudal region, accompanied by enhanced lymphatic clearance through iliac LNs.^122^ This redistribution implies a compensatory contribution of smLVs to maintain CSF homeostasis when cranial pathways are impaired.^122^ However, the interactions between intraspinal tumors and smLVs are poorly understood. Whether smLVs facilitate anti‑tumor immune surveillance, support antigen clearance, or inadvertently promote tumor cell dissemination remains an open question requiring systematic investigation.

Overall, available evidence supports that smLVs exert context‑dependent effects, either promoting repair and immune homeostasis or contributing to pathological remodeling.^5,26^ While research on cranial mLVs has advanced rapidly, studies of spinal lymphatics remain scarce. Limited evidence suggests a potential shift in the waste drainage pattern from CLNs to caudal region LNs following cranial or upper spinal cord pathologies, such as stroke and tumor^117,122^ (Fig. 3c). Future efforts combining high‑resolution imaging, genetic labeling, and functional assays will be essential to establish mechanistic insight into smLV roles across CNS malignancies and related spinal diseases.

## LVs in the vertebral column

### LVs in vertebra

The existence of LVs within bones has long been controversial. Historically, bone lymphatics were considered pathological structures.^31,123^ For example, Gorham-Stout disease involves abnormal proliferation of non‑neoplastic LVs, leading to osteolysis and progressive bone loss.^123^ Inhibiting LEC growth, migration, or lumen formation can mitigate osteolysis in this disease.^123,124^ Conventional two‑dimensional staining using LYVE‑1 and PDPN failed to detect LVs in healthy bone,^31,125^ reinforcing the long‑standing belief that bones were devoid of LVs except when tumor or inflammatory lesions breached the cortex and extended into surrounding connective tissue.^31,125^

**Fig. 4:**
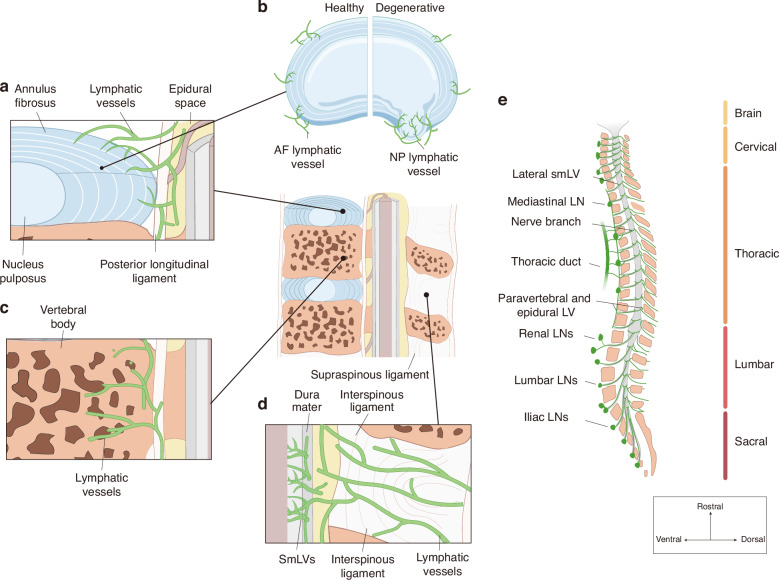
Lymphatic vessels in vertebral column and epidural/paravertebral space. **a** Distribution of lymphatic vessels (LVs) in the intervertebral disc. LVs are present in annulus fibrosus (AF), surrounding ligaments and epidural space. **b** Comparison of LV distribution in healthy and degenerated intervertebral discs (IVDs). LVs are primarily localized within the AF. Healthy IVDs contain a higher LV density, whereas degenerated discs show reduced or fragmented lymphatic structures in AF. However, the herniated nucleus pulposus (NP) exhibits a higher density of LVs. **c** Distribution of LVs in vertebral bone. LVs are present in both cortical and marrow compartments, with greater density in cortical regions. **d** Organization of epidural and paravertebral LVs in relation to the dorsal dura mater. A prominent dorsal lymphatic network extends through adjacent ligaments, where meningeal LVs connect with epidural and paravertebral branches. **e** Lateral view illustrating lymphatic vessels (LVs) extending from the spinal cord and along nerve branches to peripheral lymphatics. Spinal meningeal lymphatic vessels connect to the dorsal and ventral paravertebral LVs, which in turn drain to the corresponding lymph nodes (LNs) and duct. LV lymphatic vessel, IVD intervertebral disc, LN lymph node

Subsequent evidence, however, revealed potential links between bone marrow and the lymphatic system. Tracer studies showed that Indian ink or macromolecules such as ferritin and horseradish peroxidase injected into the marrow later appear in LNs.^126–129^ LEC markers have also been detected in the outer periosteum of vertebrae.^125^ Recent advances in tissue clearing and three‑dimensional imaging, particularly LSFM, have provided direct visualization of intrabony lymphatic networks.^28^ Biswas et al. demonstrated the presence of LVs within both cortical and marrow compartments of intact murine skeletal elements, including vertebrae, femurs, and tibiae^28^ (Fig. 4c). Vertebral LVs display striated or clustered morphologies and are more abundant than those in long bones, consistent with higher Prox1 expression in vertebral tissue.^28^ These findings suggest a richer and more complex lymphatic architecture in vertebrae than previously recognized. Future studies using LSFM, confocal microscopy, and high‑resolution 3D reconstruction should further delineate their spatial organization and microstructural diversity.

#### Functions of vertebral LVs

Although direct experimental evidence on vertebral lymphatic function remains scarce, insights from long‑bone studies provide a useful conceptual framework. In long bones, impaired lymphatic drainage correlates with fracture non‑union,^130,131^ whereas LV depletion or VEGFR‑3 blockade delays healing and reduces osteogenesis.^28,132^ Lymphatic drainage removes damage‑associated molecular patterns, supporting osteoblast survival and bone marrow stromal cell proliferation,^32^ and promotes regeneration by expanding Myh11⁺ pericytes.^28^ Conversely, lymphatic platelet thrombosis (LPT) formation blocks fluid transport, impairs drainage, and hinders repair.^32,132^ LVs also influence hematopoiesis: under stress, IL‑6‑induced LV dilation enhances marrow activity through CXCL12 secretion from LECs.^28^

However, vertebrae differ from long bones in origin, architecture, and biomechanics, which may affect lymphatic organization and function. Derived from the sclerotome rather than the limb bud,^133,134^ vertebrae exhibit spongy trabecular structures,^135,136^ and primarily experience compressive rather than bending forces.^137^ Their fracture repair involves limited callus formation^138^ and occurs within a dense neurovascular environment.^136^ These distinctions likely impose greater demands on lymphatic drainage to resolve edema, regulate inflammation, and coordinate repair.^138,139^ Thus, vertebral LVs may perform analogous roles in fracture healing and hematopoiesis but within a distinct mechanical and developmental context requiring dedicated study.

Importantly, vertebrae reside near neural and meningeal structures, suggesting that their lymphatic function extends beyond osseous repair. Vertebral LVs may form communication pathways linking the marrow, paravertebral tissues, and meningeal compartments of the CNS.^82,84,140^ Myeloid cells within the spinal meninges originate in adjacent vertebral marrow, forming a vertebra-meninges axis.^82,84,140^ Channels between the vertebral bone and dura mater allow bidirectional exchange of molecules and cells; however, their exact nature, whether lymphatic, vascular, or perivascular, and their selectivity for different substances remain unclear.^82,129,140–142^ Nonetheless, tracer studies suggest that marrow‑derived molecules reach periosteal surfaces and draining LNs,^126–129^ supporting functional vertebral lymphatic connectivity. Evidence that bone progenitor cells promote lymphangiogenesis further underscores the close interrelationship between bone and LVs.^143^ Although studies have shown that the vertebra contains abundant LVs, their unclear distribution pattern obscures their specific functions. Moreover, LVs are observed at tumor margins after bone invasion, correlating with higher metastatic potential.^31,125^ Vertebral lymphatic routes may thus provide conduits for tumor dissemination to the CNS or distant organs.

While conclusions regarding vertebral LV function should remain cautious, their proposed roles in vertebral homeostasis, immune trafficking, and tumor progression warrant focused investigation, particularly through future studies establishing lymphatic distribution models in the vertebra. Promisingly, experimental modulation of bone lymphatics yields therapeutic benefits: anti‑PDPN antibodies that prevent LPT formation accelerate fracture repair, and transplantation of young LECs into aged mice enhances bone and hematopoietic regeneration.^28,132^ Elucidating vertebral LV biology could therefore open new avenues for treating spinal, hematopoietic, and neurological disorders.

### LVs in the intervertebral disc

The IVD is a key fibrocartilaginous structure connecting adjacent vertebrae, comprising the nucleus pulposus (NP), annulus fibrosus (AF), and cartilage endplates. Traditionally regarded as the largest avascular tissue in the human body, the IVD was also assumed to lack LVs.^125,144^ However, our recent work challenges this view, revealing the presence of LVs within the AF of healthy discs^30^ (Fig. 4a). Spatial transcriptomic analysis of human IVD samples identified canonical lymphatic markers, including *Prox1* and *LYVE-1*, in AF regions. Immunohistochemistry and immunofluorescence confirmed their expression in both human and rat specimens.^30^ Discrepancies between our results and earlier reports likely stem from methodological differences, particularly in tissue source, preservation, and imaging resolution. Unlike previous studies relying on cadaveric samples,^125,144^ our use of freshly resected surgical specimens from Hirayama disease patients preserved microstructure and antigenicity, enabling detection of fine lymphatic structures previously missed. Moreover, high-resolution imaging and optimized staining protocols likely enhanced visualization of delicate LVs otherwise undetectable in earlier studies.

In degenerated IVDs, while the presence of LVs is consistently observed, studies report divergent remodeling patterns.^29,30,144^ Our data indicate a decline in LV density in degenerated IVDs, whereas other groups observed increased lymphangiogenesis,^125,144^ particularly in herniated NP^29^ (Fig. 4b). These differences likely reflect variations in baseline tissue definition, anatomical sampling (AF vs. NP), and degeneration severity.^29,125,144^ We propose a model in which AF-resident LVs progressively regress during degeneration, while de novo lymphangiogenesis occurs in exposed NP tissue depending on the extent of annular rupture and inflammatory activation.^30^ Notably, LVs within herniated NP appear continuous with epidural and paravertebral lymphatic networks.^29^ Therefore, rigorous anatomical delineation and standardized degeneration grading are essential for reconciling these discrepancies and drawing robust conclusions.

#### Functions of LVs in IVD

Dynamic changes in LV density during degeneration imply functional involvement in both injury and repair processes.^29,30,125,144^ Experimental models show that enhanced lymphangiogenesis accelerates NP resorption and resolution of inflammation.^29^ Lymphatic activity peaks within 2 weeks post-injury and diminishes by week 4, suggesting inflammation-dependent regulation of lymphatic infiltration.^29^ This process is mediated through VEGF-C/VEGFR3 signaling, as VEGFR3 blockade suppresses lymphangiogenesis, aggravates inflammation, and delays NP resorption.^29^ Consistent with these findings, our study demonstrates that LVs in the IVD facilitate inflammatory cell drainage, mitigating local inflammation and tissue damage.^30^ Conversely, LV loss correlates with amplified inflammatory responses, emphasizing their protective role in maintaining tissue homeostasis and promoting repair.^29,30^

Collectively, these findings redefine the IVD as a lymphatic-active tissue, revising classical anatomical paradigms. Beyond structural mapping, future studies should explore additional LV functions, such as nutrient transport and interplay with angiogenesis.^30^ Importantly, modulating IVD lymphangiogenesis holds therapeutic promise. PROX1 overexpression has been shown to enhance LV formation and slow the progression of IVD degeneration. To further dissect in vivo roles, locally inducible, tissue-specific mouse models, such as *Prox1-CreER*^*T2*^*; Vegfr3*^*fl/fl*^, could be employed to precisely modulate LV activity. Local administration of 4-hydroxytamoxifen, the tamoxifen metabolites, would activate CreER recombinase specifically within the injected region, thereby avoiding systemic effects associated with lymphatic depletion.^145,146^ Moreover, localized lentiviral, hydrogel or nanoplatform-based delivery of lymphangiogenic agents (e.g., VEGF-C) or inhibitors (e.g., VEGFR3 blockade or VEGF-C traps) offers tractable strategies for therapeutic manipulation.^30,147–149^ Such targeted approaches will provide mechanistic insight and pave the way for novel LV-centered interventions in disc degeneration.

## Epidural and paravertebral LVs

As an extension of the spinal and meningeal lymphatic systems, epidural and paravertebral LVs form a crucial transitional interface connecting the central and peripheral lymphatic networks.^29,35^ These vessels reside within the epidural space and paravertebral tissues along the vertebral column, establishing a continuous drainage pathway that channels interstitial fluid, immune cells, and metabolites from the spinal cord and vertebrae toward regional LNs.^29,35^ Positioned at the convergence of neural, vascular, and skeletal compartments, they likely share core functions with peripheral lymphatics while exhibiting specialized adaptations to the spinal microenvironment.^35,48,150^ This section outlines their spatial organization, drainage patterns, and physiological and pathological roles, emphasizing their integrative function within the broader spinal lymphatic network.

### Architecture organization and drainage pathways

Epidural lymphatics primarily occupy intervertebral spaces, forming segmental loops interconnected by longitudinal LVs that run parallel to the spinal axis.^35^ Dorsally, semicircular channels connect epidural lymphatics with vessels of the dorsal plexus, passing through the ligamentum flavum and linking to peripheral lymphatics that penetrate bilateral spinous processes^35^ (Fig. 4d, e). The cervical and thoracic regions exhibit denser dorsal lymphatic plexuses than the lumbar region,^35,48^ while dorsolateral branches extend toward facet joints. At the intervertebral foramina, ventral and dorsal lymphatics envelop the DRG and connect with ventral lymphatics and regional nodes.^35^ Tracer and anatomical studies indicate that cervical lymphatics drain into deep CLNs, thoracic LVs connect directly with the thoracic duct and mediastinal nodes through paravertebral LV, and lumbar LVs bifurcate toward renal and iliac LNs^35^ (Fig. 4e). Epidural LVs also extend through connective tissues and ligaments, including the posterior longitudinal ligament,^35,125^ integrating with vertebral and intervertebral drainage systems (Fig. 4a, c, d, e).

### Functional significance and pathological relevance

Epidural and paravertebral LVs serve as efferent routes for spinal meningeal lymphatic drainage, completing the continuum from the CNS to the peripheral lymphatic circulation.^29,35^ Enhanced lymphangiogenesis within paravertebral tissues beyond the IVD improves lymphatic clearance, reducing immune cell accumulation and promoting resolution of inflammation in models of NP herniation.^29,30^ Conversely, these vessels may also mediate retrograde immune cell transport, contributing to spondyloarthritis pathogenesis through dendritic cell migration.^151^ These vessels may therefore act as dynamic regulators of spinal fluid homeostasis and inflammation.

Analogous findings in peripheral musculoskeletal systems reinforce their likely roles in maintaining spinal articular health. In joints, lymphatic expansion promotes tendon repair,^152^ while impaired contractility aggravates osteoarthritis^153,154^ and rheumatoid arthritis.^29,155^ By extension, epidural and paravertebral LVs may support clearance of inflammatory mediators and maintain joint integrity in the spine, although direct evidence remains limited.

Paravertebral LVs are also implicated in tumor biology and immune modulation. LVs frequently localize at tumor invasion margins, where they may facilitate cancer cell dissemination.^31,125^ Although spinal lymphangiomas are exceedingly rare (<40 cases),^156^ lymphatic involvement has been observed in metastatic and hematologic spinal malignancies.^157,158^

In summary, epidural and paravertebral LVs function as integral extensions of the spinal lymphatic continuum, bridging smLVs, vertebral lymphatics, and peripheral nodes. Their strategic localization enables regulation of interstitial fluid clearance, immune cell trafficking, and potentially tumor dissemination within the spine. Future research should define their structural diversity, drainage efficiency, and molecular regulation to elucidate their contributions to spinal homeostasis and disease.

## Conclusion

Recent advances have revealed that the spinal lymphatic system plays pivotal roles in cerebrospinal fluid drainage, immune regulation, and tissue homeostasis, fundamentally reshaping our understanding of spinal physiology and pathology.^26,33^ smLVs demonstrate segment-specific drainage capacity and remarkable plasticity influenced by posture, circadian rhythms, and disease states.^36,55,66,72^ Acting as dual modulators of fluid clearance and neuroimmune signaling, smLVs contribute to both protective and pathological outcomes during SCI, neuroinflammation, and neurodegeneration.^35,45,90^

Beyond the spinal cord, the identification of LVs within vertebral bone and IVDs overturns the long-standing assumption of skeletal alymphaticity.^28,30^ These findings highlight lymphatic participation in bone remodeling, hematopoiesis, and IVD maintenance.^28,30^ Moreover, emerging evidence of anatomical and functional connectivity among vertebral bone marrow, the dura mater, and paraspinal lymphatic networks supports the concept of a unified spinal lymphatic system.^35,125,141,142,144^ This integrated network likely mediates fluid and immune exchange between the CNS and peripheral compartments, potentially explaining clinical associations between spinal and neurological disorders, including cervical spondylosis with dizziness, lumbar stenosis with cognitive decline, and SCI with demyelinating diseases such as MS.

### Current challenges and future directions

Despite the breakthroughs, key questions remain unanswered. Significant technical challenges persist, including: (1) how to visualize and quantify cerebrospinal fluid outflow from segmental spinal LVs; (2) how to obtain intact, well-defined spinal tissue samples suitable for microarchitectural and molecular characterization; and (3) how to selectively target spinal lymphatic function, given that conventional systemic drug delivery minimally affects perispinal lymphatics.

To address these gaps, several methodological directions merit emphasis. High-resolution in vivo tracer imaging can map drainage dynamics and efficiency^36,54^; transmission electron microscopy can clarify lymphatic endothelial junctional morphology^13^; advanced three-dimensional imaging and multi-omics profiling may delineate molecular heterogeneity and regional specificity of spinal LVs^28,54^; and region-specific gene induction or biomaterial-based drug delivery systems may enable spatially precise modulation of spinal lymphatic function.^28,147^

Resolving these challenges will illuminate: (1) the anatomical continuity between spinal and peripheral lymphatic systems; (2) the structural-functional coupling of spinal lymphatic networks; (3) the contribution of lymphatic dysfunction to neurological and musculoskeletal disorders; and (4) the translational feasibility and safety of lymphatic-targeted interventions, such as selective modulation of the VEGF-C/VEGFR-3 or PROX1 signaling pathways, which may be beneficial for bone regeneration, slowing IVD degeneration, and promoting recovery from neurological disorders. Ultimately, advancing our mechanistic understanding of spinal lymphatic biology will redefine CNS-peripheral communication and support the rational design of targeted, lymphatic-based therapies for spinal and neurological diseases.
